# A RELIABLE AND VALID ASSESSMENT OF UPPER LIMB MOVEMENT QUALITY AFTER STROKE: THE OBSERVATIONAL DRINKING TASK ASSESSMENT

**DOI:** 10.2340/jrm.v56.40362

**Published:** 2024-10-09

**Authors:** Minnu JOSE, Maria MUNOZ-NOVOA, Margit ALT MURPHY

**Affiliations:** 1 University of Gothenburg, Institute of Neuroscience and Physiology, Clinical Neuroscience, Gothenburg; 2 Department of Occupational Therapy and Physiotherapy, Sahlgrenska University Hospital, Gothenburg; 3 University of Gothenburg, Institute of Neuroscience and Physiology, Health and Rehabilitation, Gothenburg, Sweden

**Keywords:** compensatory movement strategies, hemiparesis, kinematics, movement quality, observational assessment, reliability, validity, upper limb

## Abstract

**Objective:**

To develop and evaluate the reliability and validity of a new observational Drinking Task Assessment (DTA) designed to assess quality of movement in task performance after stroke.

**Design:**

Reliability and validity.

**Methods:**

The DTA measures movement time and movement quality (smoothness, trunk, shoulder, elbow, and grasp movements) on a 4-level ordinal scale. Thirty participants with chronic stroke were assessed independently by 2 therapists. Intra-class correlation (ICC), standard error of measurement (SEM) and minimal real difference (MRD), weighted kappa, percentage of agreement, and Svensson method were used for reliability assessment. Motion capture-based kinematics and established clinical scales were used to evaluate validity.

**Results:**

The absolute SEM and MRD for movement time were 0.4 and 1 s (11%), respectively. The ICC (≥ 0.93) and weighted kappa (0.71–1.0) showed good to excellent agreement for intra- and inter-rater reliability. DTA showed strong correlations with Fugl–Meyer Assessment (0.74), Action Research Arm Test (0.93), and kinematic measures of smoothness (0.93), trunk displacement (0.91), elbow extension (0.73), and shoulder movements (0.56), indicating good construct validity.

**Conclusions:**

The new DTA proved to be a reliable and valid tool for assessment of movement quality during task performance after stroke.

## LAY ABSTRACT

Assessing movement quality is essential for understanding and monitoring recovery after stroke. However, advanced motion capture systems that provide precise analysis of movement patterns are not commonly available in clinical settings. To meet this clinical need, we developed the Drinking Task Assessment and evaluated its reliability and validity. We used a rigorous iterative process including feedback from clinical experts. The results showed that the Drinking Task Assessment has strong measurement properties and can effectively assess upper limb movement quality in people who have had a stroke. The scale will serve as a valuable alternative when advanced kinematic analysis is not feasible or necessary and thereby fill an important gap in clinical practice.

Stroke remains a leading cause of disability and a significant burden on society worldwide (1). About half experience impairments of the upper limb early after stroke (2, 3) and most have residual upper limb impairments limiting independence in activities of daily living (ADL) and participation in major life activities (4–6).

Upper limb function is essential for various daily tasks that require the ability to reach, grasp, and manipulate different objects in an everyday context. Previous research using kinematic analysis in people with stroke has demonstrated that movements of the affected arm are slower, less precise, and less smooth (7–10). The use of alternative compensatory movement strategies is also commonly seen after a stroke. Typically, excessive shoulder elevation and arm abduction combined with forward movement of the trunk during reaching is used to compensate for decreased ability to stabilize the shoulder girdle and to produce enough voluntary elbow extension and shoulder flexion (7, 11–14). The habitual use of compensatory movement patterns might also impede the recovery of motor functions that would allow more optimal and efficient movement performance (15).

Rehabilitation of the upper limb aims to minimize sensorimotor impairments and promote arm use in ADL (12, 16, 17), but reliable and validated outcome measures addressing the qualitative aspects of movement performance during functional tasks are lacking (18–20). Assessment of movement quality is crucial for stroke rehabilitation, as it provides an insight into neurological recovery mechanisms and allows differentiation between behavioural restitution and compensation (17, 18). A standardized evaluation of movement will also enhance clinical decision-making so that appropriate treatment solutions can be selected for the specific patient.

High-speed 3D motion capture analysis is the recommended method for the measurement of movement performance and quality in people with stroke (18). These systems are, however, expensive, require expertise, and are not readily available or feasible in every clinical setting. A clinical observation-based tool addressing movement quality, specifying the key elements of the task performance, would provide an alternative tool to more advanced laboratory-based systems (21–23).

Previously, the observation-based Reaching Performance Scale (RPS) was developed for people with stroke to evaluate the quality of movement when reaching for close and far targets (23). The specific constraints, such as grasping and manipulation of a glass and the functional goal of the drinking task, are different compared with reaching for a cone. Therefore, a tool assessing movement quality during a more ecologically valid purposeful task is needed. The drinking task is already established and recommended for kinematic analysis in people with stroke (18), which makes it a good reference for validity. Thus, this study aims to develop and evaluate the reliability and validity of a new observational Drinking Task Assessment (DTA) designed to assess the quality of movement in task performance in people with stroke.

## METHODS

This study was approved by the Swedish ethical review authority (2022-00443-02) and prior written informed consent was obtained from all participants. The COSMIN (Consensus-Based Standards for the Selection of Health Measurement Instruments) principles were followed (24). The funders played no role in the design, conduct, or reporting of this study.

### Development of the Drinking Task Assessment

An expert group, including 3 physiotherapists (a senior researcher with 15 years and 2 junior researchers with 2 years’ clinical experience in stroke rehabilitation) developed the observational DTA through an iterative process. The RPS was used as a starting template (23). The selection of movement components (items) of the DTA was guided by knowledge gained from kinematic analysis of the drinking task (7, 25) along with clinical expertise of the expert group. The drafted versions of the DTA were tested in clinical settings by 5 external experienced clinicians (3 physiotherapists and 2 occupational therapists) working with stroke rehabilitation to provide feedback. The English and Swedish versions were drafted in parallel, through forward and back translations by an official authorized translator and consensus discussions within the expert group and with external clinicians to ensure relevance to clinical practice and semantic equivalence between the 2 versions.

The DTA comprises 6 key components: movement time, movement smoothness, trunk displacement, shoulder flexion, elbow extension, and grasping, graded on a 4-grade scale, resulting in a sum score of 18 points. In addition, a global score ranging from 0 to 5 is used to rate overall performance. The component score and the global score are summed to a total score of 23 points, which indicates performance comparable to persons without upper limb impairments. It takes about 5 to 10 min administration time to perform the DTA and complete the scoring. The final DTA protocol with instructions, see Appendix S1.

### Participants

This cross-sectional study included a convenient sample of 30 individuals, recruited through stroke rehabilitation centres and patient organizations in the Gothenburg area, Sweden, between June 2022 and January 2023. The inclusion criteria were: adults with chronic stroke, having a residual upper limb impairment due to stroke, defined as less than a maximum of 66 points om the Fugl–Meyer Assessment of Upper Extremity. Participants were excluded from the study if they were unable to follow instructions needed for the assessments and/or presented another upper limb condition affecting upper limb movement performance.

### Reliability and validity assessment

For the inter-rater reliability, 2 trained raters (MAM and MJ) recorded the movement time and scored all items of the DTA independently during the assessment session. The task performance was also videotaped from 2 different angles (side and front). Movement time was manually recorded by a digital stopwatch. The stopwatch was started when the hand started to move from the initial position on the table and stopped when the hand was back and stationary in the initial position. A mean of 3 trials was calculated as the final movement time. For the intra-rater reliability, the recorded assessments were assessed from videotapes on 2 separate occasions with a month’s break between. Concurrent validity of the DTA was established to standardized upper extremity rating scales, to patient-reported scales, and to specific variables of the kinematic data (described in detail below).

### Procedures for the drinking task

**Fig. 1:**
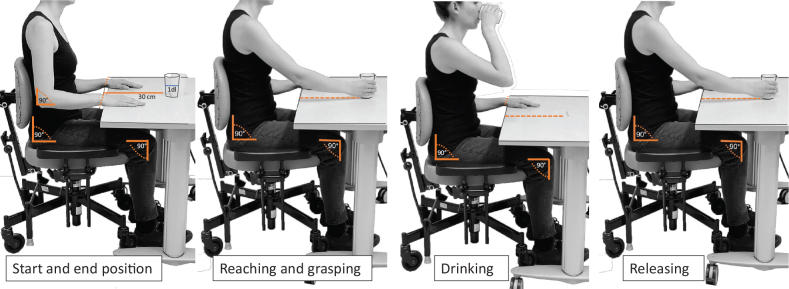
Standardized sitting position and movement phases of the drinking task.

A standardized established kinematic analysis protocol of the drinking task was used (7, 25). The drinking task included 5 movement phases: reaching for the glass, moving the glass to the mouth, drinking a sip of water, moving the glass back onto the table, and returning the hand to the initial position (Fig. 1) (7, 25). In the starting sitting position, the height of the chair and table was adjusted to ensure a 90-degree angle of the knee and the hip, upper arm vertical, and forearm in a horizontal position. The wrist joint was aligned with the edge of the table, the palm resting on the table. A hard plastic glass filled with 100 mL of water was placed 30 cm away from the edge of the table in the midline of the body. A few familiarization trials were performed before test trials to ensure that the participant understood the instructions. The task was repeated with natural speed 5 times with the unaffected arm first followed by the affected arm, but only 3 middle trials were used in the analysis. The mean of 3 middle trials has been shown to be sufficient to produce stable results and reach good test–retest reliability (26). A mean of the same 3 trials, measured by kinematics and the manual stopwatch, was calculated. The interval between each trial was about 5 s with 1 min between the arms.

### 3D kinematic analysis of the drinking task

A 5-camera 3D kinematic motion capture system (MCU240 Hz, Qualisys AB, Gothenburg, Sweden) was used (7, 25). The spherical reflective markers were attached to the bony anatomical prominences of the body with double-sided tape (third metacarpophalangeal joint on both arms, styloid process of ulna, lateral epicondyle of humerus, middle part of the acromion on both sides, upper part of the sternum, and notch between the eyebrows); 1 marker was attached on the upper edge of the drinking glass. The motion capture data were transferred to Matlab software (The Mathworks Inc, Natick, CA, USA) for custom-made analysis and filtered with a 6 Hz second-order Butterworth filter in both forward and backward directions, resulting in zero-phase distortion and fourth-order filtering (7, 25).

### Kinematic variables

The movement time was computed from the hand marker with defined start and stop at 2% threshold of the maximum velocity (7, 25). Movement smoothness was defined by the number of movement units (≥ 20 mm/s amplitude and ≥ 150 ms between peaks) during the 4 movement phases (except the drinking phase). The minimal number of movement units for the drinking task is 4, 1 for each phase. Trunk displacement was determined by the maximum displacement of the sternum marker during the entire task in the sagittal plane. The maximum elbow extension was extracted from the reaching phase, and shoulder abduction from the reaching and drinking phase (7, 25, 27).

### Clinical assessments

The Action Research Arm Test (ARAT) assesses upper extremity activity capacity and includes 19 items organized under 4 subscales: grasp, grip, pinch, and gross movement (28). Each item is scored on a 4-point ordinal scale and a total score of 57 marks best performance. The Fugl–Meyer Assessment of Upper Extremity (FMA-UE) assesses upper extremity motor function on a 3-point ordinal scale (29). The highest score of 66 points indicates full upper limb function. Both scales have shown excellent validity and reliability and are recommended core measures for stroke (20, 28, 30–32). In addition, ABILHAND was used to assess self-perceived manual activity in performing bimanual everyday tasks (33). The scores of the 23 questions were converted from an ordinal to a linear unidimensional scale using the Rasch method and expressed as logits ranging between –6 and +6 (33). The Stroke Impact Scale (SIS) is a self-reported questionnaire assessing consequences of stroke on 8 different domains (34). In this study the hand function subscale, including 5 predominantly unimanual tasks, scored on a 5-point scale and converted to a percentage, was used.

### Statistical analysis

The analysis was performed with the software package IBM SPSS (v 25; IBM Corp, Armonk, NY, USA) and an online analysis package (http://avdic.se/svenssonsmetod.html). To evaluate the differences in movement time (measured manually by both raters or obtained from kinematic analysis), the mean difference, 95% confidence intervals, and 95% of the limits of agreement (95% LOA) were calculated. The 95% LOA was calculated as MD ± 1.96 SD. Bland–Altman plots were used to detect systematic differences. The intra-class correlation coefficient (ICC_2,1_) was used to assess consistency of measurements. The ICC values were interpreted with ICC < 0.40 indicating poor agreement, ICC between 0.40 and 0.75 fair to good agreement, and ICC > 0.75 excellent agreement (35). The standard error of measurement (SEM) was calculated as pooled SD*√(1-ICC) (36). Lower SEM values indicate more precise measurements. Minimal real difference (MRD) was calculated as SEM*1,96*√2 to quantify the smallest real difference in a measure that can be reliably detected with a 95% confidence threshold (36). SEM% shows the relative measurement error of the mean (SEM/pooled mean*100). The MRD% indicates the relative minimal real difference of the measure (MRD/pooled mean*100). An SEM% value below 10% and MRD% values below 30% are typically considered acceptable (37).

For the analysis of inter- and intra-rater reliability of the DTA weighted kappa, percentage of agreement (PA%) and a rank invariant method (Svensson’s method) specially developed for paired ordinal data was used (28, 31, 38). The same limits as for ICC were used to interpret the values of weighted kappa (35, 37). For the PA%, agreement ≥ 70 was considered satisfactory. The Svensson’s rank-based method was used to detect systematic disagreements by the relative position (RP) and relative concentration (RC) (38). Both RP and RC range from –1 to 1, and an absolute value ≥ 0.1 can indicate a systematic difference. The relative rank variance (RV) ranges from 0 to 1, and a value higher than 0.01 indicates random error. The 95% CI was used to determine statistically significant differences.

Spearman’s rank correlation coefficients were used to determine concurrent validity with other established upper limb clinical scales and the selected kinematics (smoothness, shoulder abduction, elbow extension, trunk displacement). The correlations were interpreted as negligible (< 0.2), weak (between 0.2 and 0.4), moderate (between 0.4 and 0.7), strong (between 0.7 and 0.9), and very strong (> 0.9) (39).

## RESULTS

**Fig. 2:**
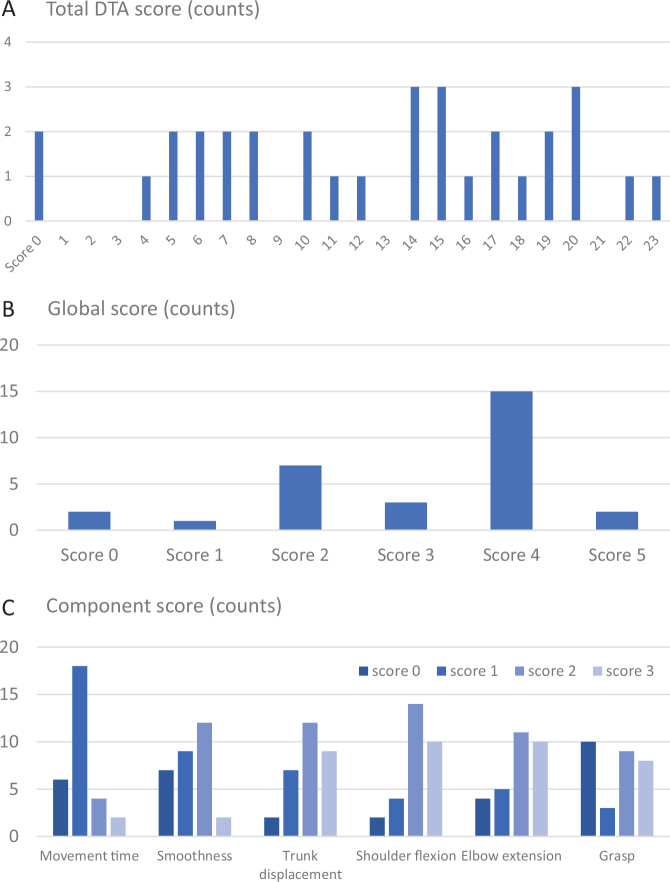
Number of participants with a specific score, shown for the (A) total score (B), global score, and (C) component scores.

Table I lists the demographic and clinical characteristics of the 30 included participants. The mean age was 61.8 years (range 42–90 years), about 67% were men and 70% had a cerebral infarct. All participants were right-handed. The sample included participants with a wide range of upper limb impairment levels (FMA-UE ranging between 9 and 62). The floor (0 score) and ceiling effects (maximum score) of the DTA were 6% and 3%, respectively. The individual scores of the included participants covered all possible scores of the component and the global score ranges (Fig. 2). Two participants were not able to complete the drinking task and received a total DTA score of zero. Three additional participants were not able to come to the assessment at the movement analysis laboratory and thus did not get any results on the kinematic measures. The final dataset included DTA scores from 30 participants, stopwatch measurements from 28 participants, and kinematic measurements from 25 participants.

**Table I:** Demographic data and clinical characteristics of the 30 participants with stroke

Male, *n* (%)	20 (66.7)
Age, mean (SD), min–max	61.8 (12.9), 42–90
Type of stroke, *n* (%)	
Cerebral infarct	21 (70)
Cerebral haemorrhage	9 (30)
Years since stroke, mean (SD), min–max	10.2 (12.1), 1–57
Paretic side left, *n* (%)	17 (56.7)
Fugl–Meyer Assessment Upper Extremity, median (IQR)	46.5 (34; 54)
Action Research Arm Test, median (IQR)	41 (30; 48)
ABILHAND, mean (SD)	2.2 (1.1)
Stroke Impact Scale hand function, mean (SD)	0.5 (0.3)
Drinking Task Assessment, median (IQR)*	
Total score (0–23)	14 (8; 18)
Global score (0–5)	4 (2; 4)
Kinematics measures of the affected arm, mean (SD), *n* = 25	
Movement time	12.1 (5.9)
Smoothness, number of movement units	20.1 (14.1)
Shoulder abduction, degrees	42.0 (13.2)
Elbow extension, degrees	64.9 (14.8)
Trunk displacement, cm	12.2 (8.9)

* Median (IQR: interquartile range) was the same for both raters (Raters A and B) on both test occasions.

### Reliability assessment of movement time

The mean differences in movement times between the raters were small (0.05–0.50 s), although a statistically significant systematic difference was noted in all but 1 comparison (Table II). The differences were smaller for the intra-rater analysis (0.05–0.13) along with a narrower 95% CI than for the inter-rater analysis.

**Table II:** Comparison of movement times in seconds of the affected and non-affected arms between 2 raters (inter-rater reliability), within the same rater (intra-rater reliability) and between manual stopwatch and the kinematic measurement

Factor	MD	SD	95% CI	95% LOA	ICC	SEM	SEM%	MRD	MRD%
Stopwatch MT (*n* = 28)									
Inter-rater, Occasion 1									
Affected arm	0.43	0.33	**0.30; 0.56**	–0.22; 1.07	0.99	0.28	2.08	0.78	5.75
Non-affected arm	0.50	0.22	**0.41; 0.58**	0.07; 0.93	0.98	0.16	2.22	0.43	6.17
Inter-rater Occasion 2									
Affected arm	0.30	0.29	**0.19; 0.41**	–0.27; 0.86	0.99	0.28	2.08	0.78	5.75
Non-affected arm	-0.24	0.29	**0.13; 0.36**	–0.33; 0.82	0.96	0.21	2.95	0.58	8.19
Intra-rater, Rater A									
Affected arm	-0.05	0.12	**0.00; 0.09**	–0.20; 0.29	1	0.00	0.00	0.00	0.00
Non-affected arm	-0.13	0.17	**0.06; 0.19**	–0.21; 0.46	0.98	0.12	1.66	0.33	4.61
Intra-rater, Rater B									
Affected arm	0.08	0.48	–0.27; 0.10	–1.03; 0.87	0.99	0.28	2.11	0.78	5.85
Non-affected arm	0.13	0.25	**–0.22; –0.03**	–0.61; 0.36	0.97	0.17	2.55	0.48	7.08
Kinematics MT, *n* = 25									
Rater A, Occasion 1									
Affected arm	0.13	0.32	**0.00; 0.26**	–0.49; 0.75	0.99	0.18	1.52	0.51	4.21
Non-affected arm	0.21	0.31	**0.08; 0.34**	–0.39; 0.81	0.96	0.22	3.06	0.60	8.49
Rater A, Occasion 2									
Affected arm	-0.07	0.34	–0.07; 0.21	–0.60; 0.74	0.99	0.26	2.16	0.72	5.98
Non-affected arm	-0.07	0.26	–0.03; 0.18	–0.44; 0.59	0.97	0.19	2.64	0.52	7.31
Rater B, Occasion 1									
Affected arm	-0.28	0.41	**–0.45; –0.11**	–1.09; 0.52	0.99	0.26	2.21	0.73	6.12
Non-affected arm	-0.28	0.33	**–0.41; –0.14**	–0.92; 0.36	0.95	0.23	3.40	0.65	9.41
Rater B, Occasion 2									
Affected arm	0.24	0.53	**–0.46; –0.02**	–1.27; 0.79	0.99	0.37	3.09	1.02	8.56
Non-affected arm	0.16	0.39	–0.32; 0.00	–0.93; 0.60	0.93	0.28	3.99	0.77	11.05

MD: mean difference; SD: standard deviation of MD; 95% CI: 95% confidence interval; ICC: intraclass correlation coefficient; SEM: standard error of measurement; MRD: minimal real difference; MT: movement time. The statistically significant differences between assessments are marked in bold.

In the comparisons between the manual and kinematic measurement of movement time, the detected systematic differences were small (mean difference 0.13–0.28). The 95% LOA values were narrow compared with mean difference (all absolute values below or close to 1 s) and symmetrically spread over the scale range around the mean difference with only a few values outside the limits in the Bland–Altman plots. The calculated SEM for the more affected arm varied between 0 and 0.37 s (maximum of 3.1%) and the MRD varied between 0 and 1.1 s (maximum of 8.6%). The calculated SEM for the less affected arm varied between 0.12 and 0.28 s (maximum of 4.0%) and the MRD varied between 0.3 and 0.8 s (maximum of 11.1%). These results confirm that approximately a 1-s difference in movement time of the more-affected arm indicates a real difference, larger than the measurement error, for the drinking task. The ICC values were very high (> 0.93), which signifies an excellent degree of agreement.

### Reliability assessment of DTA

The weighted kappa and percentage of agreement values were all above 0.71 and many reached above 0.9, indicating good to excellent agreement both for the inter-rater and intra-rater agreement (Tables III and IV). Among all items of the DTA, the lowest agreement was observed for the shoulder flexion and smoothness items, with weighted kappa of 0.72 and 0.71, respectively. For the component score and total score of the DTA a 1-point difference between the raters was sufficient to reach a PA above 80% agreement. Svensson’s method revealed statistically significant systematic disagreement in relative position for smoothness on test occasion 2 (inter-rater), in Rater B (intra-rater), and in grasp item on occasion 1 (inter-rater). A tendency towards a non-negligible disagreement in relative concentration was found for shoulder flexion on test occasion 1 (inter-rater). A tendency towards a non-negligible disagreement in relative concentration was found for shoulder flexion on test occasion 1 (interrater) (Table III). There were no random errors detected (RV< 0.1).

**Table III:** Inter-rater agreement between the rates for the global score, each item. summed component, and total scores of the Drinking Task Assessment (*n* = 30)

Occasion	Weighted kappa (95% CI)	PA%	RP (95% CI)	RC (95% CI)
Inter-rater agreement on occasion 1					
Global score	0.92 (0.84; 1.01)	90	–0.02 (–0.08; 0.04)	0.02 (–0.09; 0.14)
Movement time	0.91 (0.81; 1.02)	93	0.03 (–0.02; 0.08)	–0.04 (–0.10; 0.01)
Smoothness	0.93 (0.84; 1.02)	93	0 (–0.04; 0.04)	0 (–0.10; 0.10)
Trunk displacement	0.96 (0.89; 1.03)	97	–0.02 (–0.07; 0.02)	0.03 (–0.03; 0.09)
Shoulder flexion	0.72 (0.53; 0.90)	73	0.01 (–0.11; 0.14)	–0.11 (–0.25; 0.04)^a^
Elbow extension	0.81 (0.67; 0.94)	80	0.07 (–0.01; 0.15)	0 (–0.13; 0.13)
Grasp	0.82 (0.69; 0.96)	80	0.11 (0.02; 0.19)*	–0.08 (–0.22; 0.07)
Component score (± 1/ ± 2)	0.86 (0.81; 0.91)	37 (90/100)	0.04 (0.01; 0.08)	–0.02 (–0.10; 0.05)
Total score (± 1/ ± 2)	0.87 (0.83; 0.92)	33 (87/100)	0.03 (–0.002; 0.07)	–0.02 (–0.09; 0.06)
Inter-rater agreement on occasion 2					
Global score	0.90 (0.80; 0.99)	87	0.002 (–0.07; 0.07)	0.01 (–0.13; 0.15)
Movement time	0.91 (0.81; 1.02)	93	0.03 (–0.02; 0.08)	–0.04 (–0.10; 0.01)
Smoothness	0.71 (0.55; 0.87)	70	–0.17 (–0.27; –0.07)*	0.05 (–0.13; 0.24)
Trunk displacement	0.96 (0.89; 1.03)	97	0.02 (–0.02; 0.06)	0.02 (–0.02; 0.07)
Shoulder flexion	0.75 (0.58; 0.93)	77	0.02 (–0.09; 0.12)	0.03 (–0.11; 0.16)
Elbow extension	0.90 (0.80; 1.003)	90	0.04 (–0.01; 0.09)	0.08 (–0.01; 0.17)
Grasp	0.97 (0.92; 1.02)	97	0.02 (–0.02; 0.056)	–0.04 (–0.10; 0.03)
Component score (± 1/ ± 2)	0.90 (0.85; 0.94)	50 (93/100)	–0.004 (–0.04; 0.03)	0.03 (–0.05; 0.10)
Total (± 1/ ± 2)	0.89 (0.85; 0.93)	43 (87/100)	–0.002 (–0.04; 0.03)	0.04 (–0.04; 0.12)

* Statistically significant non-negligible disagreement (RP/RC absolute value ≥ 0.1 and 95% CI do not cover zero).

a Tendency towards a non-negligible disagreement (RP/RC absolute value ≥ 0.1 with an asymmetric 95% CI around zero). For the Component score and Total score, PA% allowing 1-point difference between rates is also shown in parentheses.

PA: percentage of agreement; RP: relative position; RC: relative concentration.

**Table IV:** Intra-rater agreement between the rates for the global score, each item, summed component, and total scores of the Drinking Task Assessment (*n* = 30)

Factor	Weighted kappa (95% CI)	PA%	RP (95% CI)	RC (95% CI)
Intra-rater A				
Global score	0.92 (84; 1.01)	90	–0.02 (–0.09; 0.05)	0.02 (–0.06; 0.11)
Movement time	1 (1; 1)	100	0	0
Smoothness	0.90 (0.79; 1.01)	90	0.06 (–0.006; 0.13)	–0.07 (–0.16; 0.01)
Trunk displacement	0.96 (0.89; 1.03)	97	–0.02 (–0.06; 0.02)	–0.02 (–0.07; 0.02)
Shoulder flexion	0.82 (0.66; 0.97)	83	–0.01 (–0.12; 0.09)	–0.08 (–0.19; 0.04)
Elbow extension	0.93 (0.85; 1.02)	93	–0.04 (–0.10; 0.01)	0.02 (–0.05; 0.09)
Grasp	0.92 (0.84; 1.007)	90	0.05 (–0.01; 0.11)	–0.08 (–0.17; 0.02)
Component (accept ± 1/ ± 2)	0.92 (0.88; 0.96)	60 (96/100)	0.01 (–0.02; 0.04)	–0.04 (–0.08; 0)
Total score (accept ± 1/ ± 2)	0.92 (0.88; 0.96)	57 (93/100)	0.01 (–0.02; 0.03)	–0.04 (–0.08 0)
Intra-rater B				
Global	0.95 (0.88; 1.01)	93	0.01 (–0.05; 0.06)	0.01 (–0.08; 0.10)
Movement time	1 (1; 1)	100	0	0
Smoothness	0.73 (0.56; 0.89)	73	–0.12 (–0.22; –0.01)*	–0.03 (–0.20; 0.14)
Trunk displacement	0.89 (0.77; 1.01)	90	0.02 (–0.05; 0.09)	–0.03 (–0.11; 0.05)
Shoulder flexion	0.72 (0.52; 0.92)	77	–0.01 (–0.14; 0.12)	0.05 (–0.07; 0.18)
Elbow extension	0.83 (0.69; 0.97)	83	–0.07 (–0.16; 0.02)	0.09 (–0.03; 0.22)
Grasp	0.87 (0.75; 1)	87	–0.03 (–0.11; 0.04)	–0.04 (–0.16; 0.08)
Component (accept ±1/±2)	0.87 (0.79; 0.94)	53 (87/93)	–0.04 (–0.09; 0.01)	0.01 (–0.08; 0.08)
Total score (accept ±1/±2)	0.88 (0.82; 0.94)	50 (83/93)	–0.03 (–0.07; 0.02)	0.01 (–0.07; 0.07)

* Statistically significant non-negligible disagreement (RP/RC absolute value ≥ 0.1 and 95% CI do not cover zero); 0 value, CI could not be calculated by the asymptotic method. For the Component score and Total score, PA% allowing 1-point difference between rates is also shown in parentheses.

PA: percentage of agreement; RP: relative position; RC: relative concentration.

### Validity assessment of DTA

Correlations between the DTA total score and motor function assessed by FMA-UE (*r* = 0.74) and ARAT (*r* = 0.93) were strong and very strong, respectively (Table V). Correlations with ABILHAND and SIS-hand function domain were moderate (*r* = 0.46). DTA global score showed similar correlations to the clinical scales, although slightly lower for the ABHILHAND (*r* = 0.35) (Table V). Correlations between specific item scores of the DTA and the kinematic measures were very strong for smoothness (*r* = –0.93) and trunk displacement (*r* = –0.91) (Table V). Strong correlation was found for elbow extension (*r* = –0.73) and moderate for shoulder abduction (*r* = 0.56), indicating that these items of the DTA reflect well the compensatory movements measured by the kinematic analysis. Kinematic analysis did not provide any comparable measure of grasp function item, although correlation between the DTA grasp item and the summed score of all grasp, grip, and pinch items of the ARAT was strong (*r* = 0.86, Table V).

**Table V:** The Drinking Task Assessment (DTA) correlations with clinical assessments and kinematic measures (*n* = 30)

Factor	Spearman correlation coefficient (CI)
DTA Total Score (0–23)	
Fugl–Meyer Assessment of Upper Extremity	0.74 (0.48–0.88)
Action Research Arm Test	0.93 (0.84–0.97)
ABILHAND	0.46 (0.10–0.71)
Stroke Impact Scale – Hand subscale	0.46 (0.10–0.71)
DTA Global score (0–5)	
Fugl–Meyer Assessment of Upper Extremity	0.77 (0.53–0.90)
Action Research Arm Test	0.91 (0.79–0.96)
ABILHAND	0.35 (–0.02–0.64)
Stroke Impact Scale – Hand subscale	0.41 (0.04–0.68)
DTA Grasp Item (0–3)	
ARAT 0–48 points (excluding gross motor sub-scale)	0.86 (0.69–0.94)
DTA Item scores, correlations with kinematics (*n* = 25)	
Smoothness	–0.93 (–0.82; –0.97)
Shoulder abduction	–0.56 (–0.18; –0.79)
Elbow extension	–0.73 (–0.43; –0.89)
Trunk displacement	–0.91 (–0.77; –0.97)

DTA: Drinking Task Assessment; ARAT: Action Research Arm Test.

## DISCUSSION

The findings of the current study showed that the newly developed scale, the Drinking Task Assessment (DTA), was reliable and valid for the assessment of movement performance and movement quality in people with stroke. Good to excellent inter- and intra-rater reliability was proven. The absolute difference in movement time, measured manually by a stopwatch or automatically by kinematic motion capture, revealed a minimal real difference of 1 s. The strong correlations with established upper limb clinical scales and the kinematic measures confirm the scale’s ability to assess qualitative differences in movement performance in people with hemiparesis after stroke. The DTA can fill the existing gap in clinical practice and research by providing a valid and reliable tool for evaluation of movement quality, thereby targeting a core question in stroke recovery, the necessity to develop better instruments to differentiate between behavioural restitution and compensation.

The observational DTA fills an important gap by providing a clinical tool for assessment of specific elements of upper limb movement quality during an ecologically valid purposeful daily task. The qualitative quantification of compensatory movement patterns is a necessity to better understand and monitor motor recovery patterns after stroke (17, 18, 23). The DTA can preferably be used when the more advanced kinematic analysis is not feasible, e.g., for patient evaluations in clinical settings or in research trials at the acute stage of stroke or when travelling to a laboratory is not a viable option for the participants. The administration burden of the DTA is low, procedures are well standardized and can be administered by any physiotherapist, occupational therapist, or health professional with equivalent training in any setting including the home. The observation of performance can be done directly but, if feasible, we recommend video recordings to allow later confirmation of scores. This provides a more secure assessment in case of any uncertainties with time taking or scoring (40). In clinical settings, the scoring of the DTA components can also completed by asking the person to make extra trials after the 3 timed trials. Assessment from the videos makes the DTA also a suitable tool for remote assessment.

The components of the DTA were carefully selected considering the extensive knowledge and established recommendations for upper limb kinematic analysis (18). The components and scoring levels of the established RPS were used as starting template for the DTA (23). However, substantial adaptations were made as the functional goal and constraints of the drinking task differ from the reaching for and grasping of a cone as quickly as possible. In addition, the global score developed for DTA expands the scoring to the lower end of the movement performance by allowing task completion with modifications, e.g., with some help from the other hand. In this way, the DTA can be introduced even when the task cannot be completed unimanually, e.g., early after stroke, which provides for wider application and monitoring over time.

The reliability analysis demonstrated excellent agreement both for the total and global scores (weighted kappa > 0.86). The intra-rater agreement was excellent for all components scored by rater A (experienced rater), while rater B showed minor systematic differences on 2 items. For the smoothness, rater B had systematically selected lower scores on the second test occasion, which also resulted in systematic differences between the raters on the second test occasion. For the grasp item, systematic discrepancies were observed between the raters on the first but not the second occasion. The raters were purposefully selected to represent a valid situation commonly seen in clinical settings, where both experienced and novice assessors can perform the scorings.

For both research and clinical settings, it is useful to know the approximate measurement error of the scale. Our results showed only minor absolute disagreements between the raters for the global and component scorings (PA ≥ 70%). For the total score the sufficient agreement (PA > 83%) was reached when a 1-point difference between the rates was accepted. This means that the estimated measurement error for the DTA total score is approximately 1 point (4.3%) and with a 2-point difference (8.7%) almost full agreement is reached (PA between 93% and 100%). Direct comparison of our reliability estimates with the RPS are difficult, as only parametric statistical estimates (ICC, SEM, and MDC) were reported for RPS, despite the ordinal nonparametric scorings (41). The minimal detectable change (MDC), indicating the minimal real difference larger than the measurement error for the RPS with a maximum score of 18 points, was reported to be 2.46 (18%) for the close target (41). Thus, our reliability analysis of the DTA showed that the measurement error could be estimated to be in the same range or even lower than reported for the RPS.

Within the DTA, the movement time is included in the total score as a categorical score. However, the exact measured movement time can also be used separately, similar to other timed tests, such as the Nine Hole Peg Test, Box and Block test, or 10 metres walking test. Here, the reference values for men and women for different age groups are also available from kinematic analysis (18), which increases the clinical utility of the movement time measurement. In the current study, the absolute MRD for movement time measured manually by a stopwatch and compared with the other rater or with kinematics was determined to be approximately 1 s. The relative MRD computed for the more affected arm was 8.6 %, which is significantly smaller than the typically accepted 30% in stroke populations (42, 43). Our MRD was also lower compared with other timed tests, such as the Nine Hole Peg Test (24–32 %) and Box and Block Test (16%) (37, 44). The MRD for the movement time computed from kinematic analysis of the drinking task has been reported to be approximately 6.4% in non-disabled controls (45). The MDC% can vary significantly (28–42%) in different kinematic studies in people with stroke, most likely due to differences in task constraints, set-ups, and analysis methods (46, 47). In the current study we demonstrated much smaller values for movement time measurement errors, with all values below 10% cut-offs for the more-affected arm.

The DTA correlated strongly with established clinical assessments of FMA-UE (0.74) and ARAT (0.93). The high correlation with ARAT can be linked to the dominance of grasp-related items in this scale, while the FMA-UE includes larger proportion of items linked to arm movements more generally. The self-reported scales, ABILHAND, and SIS Hand showed moderate correlations with the DTA, which shows the potential discrepancies between observed and self-reported measures (48). Correlations were similar for the DTA global score, confirming its validity even when used alone. Correlations with specific kinematic measures were strong for smoothness, trunk displacement, and elbow extension (0.73–0.93) and moderate for shoulder abduction (0.56). The strong correlations of specific items of the DTA with kinematic measures of smoothness, shoulder, elbow, and trunk movements, strengthens the scale’s ability to assess qualitative differences in movement performance with good accuracy. Our results are well in line with previous studies (22, 41). For example, ARAT correlated with kinematic measures of the drinking task, e.g., smoothness (*r* = 0.81), movement time (*r* = 0.68) and trunk displacement (*r* = 0.63), while correlations between kinematics and ABILHAND were weaker (*r* = 0.80–0.37) (27). Similarly, the kinematic measures of trunk displacement, elbow extension, and smoothness explained 47% of the variance in the close target RPS (41).

### Strengths and limitations

A rigorous iterative process, including feedback from clinical experts, was used to develop and adapt the key components and the scoring levels for the DTA. The participants who were included represented a wide range of upper limb impairments, covering all categories of the scales. These aspects strengthen the construct validity of the developed DTA for stroke populations. The additional low categories (0–2 points) of the DTA global score provide a possibility to use the scale in individuals with poor grasp function, early after stroke, or when an improvement is expected over time. Our results also confirmed that the DTA global score has sufficient reliability and validity to use it as a short assessment of movement quality after stroke. However, like any observational scale, the DTA is dependent on the observers’ experience and due to its ordinal nature is less precise than for example a ratio-level kinematic measurement. Our results show that the assessments can be more consistent when a more experienced assessor is using the DTA, which underscores the importance of including some prior training to reach the highest reliability. Moreover, future studies need to evaluate the responsiveness of the DTA at different stages of stroke recovery to fully evaluate the psychometric properties of this new scale.

### Conclusions

The Drinking Task Assessment is a reliable and valid scale for observational assessment of upper limb movement performance and quality in people with stroke. The scale can be a valuable alternative for assessment when a more advanced kinematic analysis is not clinically feasible or required. A detailed standardized protocol for the DTA is available with the estimated values for minimal real difference. The strong correlation with ARAT, FMA, and kinematics confirms sufficient concurrent and construct validity of the DTA in stroke populations.

## Supplementary Material

A RELIABLE AND VALID ASSESSMENT OF UPPER LIMB MOVEMENT QUALITY AFTER STROKE: THE OBSERVATIONAL DRINKING TASK ASSESSMENT

## Data Availability

The data supporting the findings of this study are available via corresponding author on reasonable request.

## References

[1] Global, regional, and national burden of stroke and its risk factors, 1990–2019: a systematic analysis for the Global Burden of Disease Study 2019. Lancet Neurol 2021; 20: 795–820. 34487721 10.1016/S1474-4422(21)00252-0 PMC8443449

[2] Persson HC, Parziali M, Danielsson A, Sunnerhagen KS. Outcome and upper extremity function within 72 hours after first occasion of stroke in an unselected population at a stroke unit: a part of the SALGOT study. BMC Neurol 2012; 12: 162. 10.1186/1471-2377-12-162 23273107 PMC3554428

[3] Simpson LA, Hayward KS, McPeake M, Field TS, Eng JJ. Challenges of estimating accurate prevalence of arm weakness early after stroke. Neurorehabil Neural Repair 2021; 35: 871–879. 10.1177/15459683211028240 34319189 PMC8442135

[4] Hartman-Maeir A, Soroker N, Oman SD, Katz N. Awareness of disabilities in stroke rehabilitation: a clinical trial. Disabil Rehabil 2003; 25: 35–44. 10.1080/0963828021000007897 12554390

[5] Riksstroke. The Swedish Stroke Register. Annual Report 2020. [cited 2021-06-29] Available from: http://www.riksstroke.org.

[6] Nijland RH, van Wegen EE, Harmeling-van der Wel BC, Kwakkel G, Investigators E. Presence of finger extension and shoulder abduction within 72 hours after stroke predicts functional recovery: early prediction of functional outcome after stroke: the EPOS cohort study. Stroke 2010; 41: 745–750. 10.1161/STROKEAHA.109.572065 20167916

[7] Alt Murphy M, Willen C, Sunnerhagen KS. Kinematic variables quantifying upper-extremity performance after stroke during reaching and drinking from a glass. Neurorehabil Neural Repair 2011; 25: 71–80. 10.1177/1545968310370748 20829411

[8] Rohrer B, Fasoli S, Krebs HI, Hughes R, Volpe B, Frontera WR, et al. Movement smoothness changes during stroke recovery. J Neurosci 2002; 22: 8297–8304. 10.1523/JNEUROSCI.22-18-08297.2002 12223584 PMC6758113

[9] Alt Murphy M, Häger CK. Kinematic analysis of the upper extremity after stroke: how far have we reached and what have we grasped? Phys Ther Rev 2015; 20: 137–155. 10.1179/1743288X15Y.0000000002

[10] Schwarz A, Kanzler CM, Lambercy O, Luft AR, Veerbeek JM. Systematic review on kinematic assessments of upper limb movements after stroke. Stroke 2019; 50: 718–727. 10.1161/STROKEAHA.118.023531 30776997

[11] Roby-Brami A, Feydy A, Combeaud M, Biryukova EV, Bussel B, Levin MF. Motor compensation and recovery for reaching in stroke patients. Acta Neurol Scand 2003; 107: 369–381. 10.1034/j.1600-0404.2003.00021.x 12713530

[12] Thielman G, Kaminski T, Gentile AM. Rehabilitation of reaching after stroke: comparing 2 training protocols utilizing trunk restraint. Neurorehabil Neural Repair 2008; 22: 697–705. 10.1177/1545968308315998 18971384

[13] Cirstea MC, Levin MF. Compensatory strategies for reaching in stroke. Brain 2000; 123: 940–953. 10.1093/brain/123.5.940 10775539

[14] van Kordelaar J, van Wegen EE, Kwakkel G. Unraveling the interaction between pathological upper limb synergies and compensatory trunk movements during reach-to-grasp after stroke: a cross-sectional study. Experimental brain research. Experimentelle Hirnforschung. Experimentation cérébrale 2012; 221: 251–262. 10.1007/s00221-012-3169-6 22791198 PMC3412086

[15] Cirstea MC, Levin MF. Improvement of arm movement patterns and endpoint control depends on type of feedback during practice in stroke survivors. Neurorehabil Neural Repair 2007; 21: 398–411. 10.1177/1545968306298414 17369514

[16] Langhorne P, Coupar F, Pollock A. Motor recovery after stroke: a systematic review. Lancet Neurol 2009; 8: 741–754. 10.1016/S1474-4422(09)70150-4 19608100

[17] Kwakkel G, Stinear C, Essers B, Munoz-Novoa M, Branscheidt M, Cabanas-Valdes R, et al. Motor rehabilitation after stroke: European Stroke Organisation (ESO) consensus-based definition and guiding framework. Eur Stroke J 2023: 23969873231191304. 10.1177/23969873231191304 PMC10683740 37548025

[18] Kwakkel G, van Wegen EEH, Burridge JH, Winstein CJ, van Dokkum LEH, Alt Murphy M, et al. Standardized measurement of quality of upper limb movement after stroke: consensus-based core recommendations from the Second Stroke Recovery and Rehabilitation Roundtable. Neurorehabil Neural Repair 2019; 33: 951–958. 10.1177/1545968319886477 31660781

[19] Bernhardt J, Hayward KS, Dancause N, Lannin NA, Ward NS, Nudo RJ, et al. A stroke recovery trial development framework: consensus-based core recommendations from the Second Stroke Recovery and Rehabilitation Roundtable. Neurorehabil Neural Repair 2019; 33: 959–969. 10.1177/1545968319888642 31674274

[20] Pohl J, Held JPO, Verheyden G, Alt Murphy M, Engelter S, Floel A, et al. Consensus-based core set of outcome measures for clinical motor rehabilitation after stroke: a Delphi study. Front Neurol 2020; 11: 875. 10.3389/fneur.2020.00875 33013624 PMC7496361

[21] Quinn L, Riley N, Tyrell CM, Judd DL, Gill-Body KM, Hedman LD, et al. A framework for movement analysis of tasks: recommendations from the Academy of Neurologic Physical Therapy’s Movement System Task Force. Phys Ther 2021; 101: pzab154. 10.1093/ptj/pzab154 34160044

[22] Bernhardt J, Bate PJ, Matyas TA. Accuracy of observational kinematic assessment of upper-limb movements. Phys Ther 1998; 78: 259–270. 10.1093/ptj/78.3.259 9520971

[23] Levin MF, Desrosiers J, Beauchemin D, Bergeron N, Rochette A. Development and validation of a scale for rating motor compensations used for reaching in patients with hemiparesis: the reaching performance scale. Phys Ther 2004; 84: 8–22. 10.1093/ptj/84.1.8 14992673

[24] Mokkink LB, Terwee CB, Knol DL, Stratford PW, Alonso J, Patrick DL, et al. The COSMIN checklist for evaluating the methodological quality of studies on measurement properties: a clarification of its content. BMC Med Res Methodol 2010; 10: 22. 10.1186/1471-2288-10-22 20298572 PMC2848183

[25] Alt Murphy M, Murphy S, Persson HC, Bergstrom UB, Sunnerhagen KS. Kinematic analysis using 3D motion capture of drinking task in people with and without upper-extremity impairments. J Vis Exp 2018: 57228. 10.3791/57228-v PMC5933268 29658937

[26] Frykberg GE, Grip H, Alt Murphy M. How many trials are needed in kinematic analysis of reach-to-grasp? A study of the drinking task in persons with stroke and non-disabled controls. J Neuroeng Rehabil 2021; 18: 101. 10.1186/s12984-021-00895-3 34130716 PMC8207615

[27] Alt Murphy M, Willen C, Sunnerhagen KS. Movement kinematics during a drinking task are associated with the activity capacity level after stroke. Neurorehabil Neural Repair 2012; 26: 1106-1115. 10.1177/1545968312448234 22647879

[28] Nordin A, Alt Murphy M, Danielsson A. Intra-rater and inter-rater reliability at the item level of the Action Research Arm Test for patients with stroke. J Rehabil Med 2014; 46: 738–745. 10.2340/16501977-1831 24953235

[29] Fugl-Meyer AR, Jaasko L, Leyman I, Olsson S, Steglind S. The post-stroke hemiplegic patient. 1. A method for evaluation of physical performance. Scand J Rehabil Med 1975; 7: 13–31. 10.2340/1650197771331 1135616

[30] Yozbatiran N, Der-Yeghiaian L, Cramer SC. A standardized approach to performing the action research arm test. Neurorehabil Neural Repair 2008; 22: 78–90. 10.1177/1545968307305353 17704352

[31] Hernandez ED, Galeano CP, Barbosa NE, Forero SM, Nordin A, Sunnerhagen KS, et al. Intra- and inter-rater reliability of Fugl-Meyer Assessment of Upper Extremity in stroke. J Rehabil Med 2019; 51: 652–659. 10.2340/16501977-2590 31448807

[32] Alt Murphy M, Resteghini C, Feys P, Lamers I. An overview of systematic reviews on upper extremity outcome measures after stroke. BMC Neurol 2015; 15: 29. 10.1186/s12883-015-0292-6 25880033 PMC4359448

[33] Penta M, Tesio L, Arnould C, Zancan A, Thonnard JL. The ABILHAND questionnaire as a measure of manual ability in chronic stroke patients: Rasch-based validation and relationship to upper limb impairment. Stroke 2001; 32: 1627–1634. 10.1161/01.STR.32.7.1627 11441211

[34] Duncan PW, Bode RK, Min Lai S, Perera S. Rasch analysis of a new stroke-specific outcome scale: the Stroke Impact Scale. Arch Phys Med Rehabil 2003; 84: 950–963. 10.1016/S0003-9993(03)00035-2 12881816

[35] Fleiss JL, Levin BA, Paik MC. Statistical methods for rates and proportions. Hoboken, NJ: Wiley; 2003. 10.1002/0471445428

[36] Weir JP. Quantifying test–retest reliability using the intraclass correlation coefficient and the SEM. J Strength Cond Res 2005; 19: 231–240. 10.1519/00124278-200502000-00038 15705040

[37] Ekstrand E, Lexell J, Brogardh C. Test–retest reliability and convergent validity of three manual dexterity measures in persons with chronic stroke. PM R 2016; 8: 935–943. 10.1016/j.pmrj.2016.02.014 26972364

[38] Svensson E. Different ranking approaches defining association and agreement measures of paired ordinal data. Stat Med 2012; 31: 3104–3117. 10.1002/sim.5382 22714677

[39] Schober P, Boer C, Schwarte LA. Correlation coefficients: appropriate use and interpretation. Anesth Analg 2018; 126: 1763–1768. 10.1213/ANE.0000000000002864 29481436

[40] Ferrarello F, Bianchi VAM, Baccini M, Rubbieri G, Mossello E, Cavallini MC, et al. Tools for observational gait analysis in patients with stroke: a systematic review. Phys Ther 2013; 93: 1673–1685. 10.2522/ptj.20120344 23813091

[41] Subramanian SK, Banina MC, Turolla A, Levin MF. Reaching performance scale for stroke: test–retest reliability, measurement error, concurrent and discriminant validity. PM R 2022; 14: 337–347. 10.1002/pmrj.12584 33675151

[42] Chen H-M, Chen CC, Hsueh I-P, Huang S-L, Hsieh C-L. Test–retest reproducibility and smallest real difference of 5 hand function tests in patients with stroke. Neurorehabil Neural Repair 2009; 23: 435–440. 10.1177/1545968308331146 19261767

[43] Flansbjer U-B, Holmbäck AM, Downham D, Lexell J. What change in isokinetic knee muscle strength can be detected in men and women with hemiparesis after stroke? Clin Rehabil 2005; 19: 514–522. 10.1191/0269215505cr854oa 16119407

[44] Chen HM, Chen CC, Hsueh IP, Huang SL, Hsieh CL. Test–retest reproducibility and smallest real difference of 5 hand function tests in patients with stroke. Neurorehabil Neural Repair 2009; 23: 435–440. 10.1177/1545968308331146 19261767

[45] Alt Murphy M, Sunnerhagen KS, Johnels B, Willen C. Three-dimensional kinematic mot-ion analysis of a daily activity drinking from a glass: a pilot study. J Neuroeng Rehabil 2006; 3: 18. 10.1186/1743-0003-3-18 16914057 PMC1562432

[46] Patterson TS, Bishop MD, McGuirk TE, Sethi A, Richards LG. Reliability of upper extremity kinematics while performing different tasks in individuals with stroke. J Mot Behav 2011; 43: 121–130. 10.1080/00222895.2010.548422 21347950

[47] Sousa ASP, da Silva CIC, Mesquita IA, Silva A, Macedo R, Imatz-Ojanguren E, et al. Optimal multi-field functional electrical stimulation parameters for the “drinking task – reaching phase” and related upper limb kinematics repeatability in post stroke subjects. J Hand Ther 2022; 35: 645–654. 10.1016/j.jht.2021.05.002 34253404

[48] Hussain N, Alt Murphy M, Lundgren-Nilsson A, Sunnerhagen KS. Relationship between self-reported and objectively measured manual ability varies during the first year post-stroke. Sci Rep 2020; 10: 5093. 10.1038/s41598-020-61834-1 32198393 PMC7083900

