# Supplementary material for: A RELIABLE AND VALID ASSESSMENT OF UPPER LIMB MOVEMENT QUALITY AFTER STROKE: THE OBSERVATIONAL DRINKING TASK ASSESSMENT
Source: J Rehabil Med. 2024 Oct 9;56:40362. doi: 10.2340/jrm.v56.40362 (PMC11481465; doi:10.2340/jrm.v56.40362)
Supplement: A RELIABLE AND VALID ASSESSMENT OF UPPER LIMB MOVEMENT QUALITY AFTER STROKE: THE OBSERVATIONAL DRINKING TASK ASSESSMENT [file JRM-56-40362-s1.pdf]

Supplementary material has been published as submitted. It has not been copyedited, or typeset by Journal of Rehabilitation Medicine

Drinking Task Assessment - DTA

Observational assessment of movement time and quality in people with stroke

Starting position:

90º of hip and knee flexion, hand on table and the wrist in line with table edge, 90º elbow flexion, overarm in vertical and underarm in horizontal plane. Glass is placed behind a marked line 30 cm from the table edge at the body's midline (about A4 paper length). The lower back is resting against the backrest of the chair. The sitting distance from the table is defined by the vertical position of the upper arm and hand position on the table. The other hand is resting on the table.

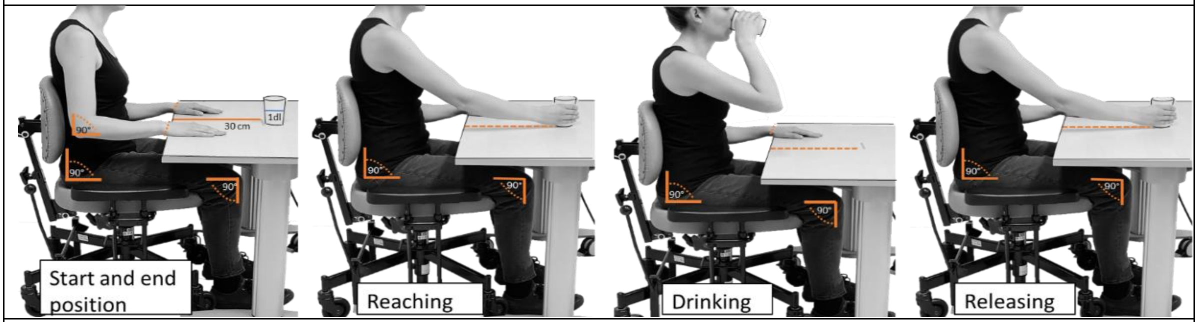

Instructions:

Sit with your back resting against the chair back. Reach and grasp the glass, move it to the mouth to take a sip of water, place the glass back behind the marked line and return the hand to the strarting position. Do the task in a natural speed as normally as possible.

Standardization:

- A drinking glass (6-7 cm diameter, 10 cm height, can be plastic) filled with 1 dl of water (about half a glass, water level can be marked as a line on the glass).
- Perform few familiarization trials to ensure that instructions are followed correctly, before taking time.
- The task is performed with the less-affected arm first, followed by the more affected arm.
- The drinking task is performed after the instruction "you can start now".
- Timing with a stopwatch starts as soon a movement of the hand is visually detected and ends when the hand is back in the starting position.
- The time is recorded for at least 3 completed trials and a mean of 3 trials is calculated (in case the first trial is slower compared to the following 2, do an extra trial and ignore the first one).
- Time is only recorded when the drinking task can be completed fully with or without minor modifications (score 2 to 5 on the Global Score on page 2).
- In presence of swallowing impairment, the tested person can wet the lips and mimic drinking instead of taking a sip of water.
- For assessment of movement quality follow the instructions on page 2 (the scoring can be done during additional non-timed trials).

Movement time

measured by a stopwatch in seconds (maximum of one decimal)

Trial number	Less-affected arm		Comments & modification	More-affected arm		Comments & modification
	Right	Left		Right	Left	
1						
2						
3						
4						
5						
6						
Mean of 3 trials						

Reference values*	**< 50 yeas**	**51-65 years**	**> 65 years**
Dominant / Non-dominant hand	5.6 ±0.8 / 5.7 ±0.8	6.3 ±0.8 / 6.3 ±0.8	6.5 ±1.0 / 6.7 ±1.1

** Alt Murphy et al. Journal of visualized experiments. March 2018; Kwakkel et al. Int J Stroke 2019;14(8):783-791.*

Drinking Task Assessment – DTA

Observational assessment of movement time and movement quality in people with stroke

Global score	
**5**	**Task is completed easily (without difficulty)**<br>Movements are smooth, continuous and no compensatory movements are observed
**4**	**Task is completed with minor difficulty**<br>Movements are e.g. not fully fluid, small compensatory trunk/arm movements, difficulties to grasp
**3**	**Task is completed with major difficulty**<br>Movements are e.g. clearly segmented, unsmooth or jerky, clear compensatory trunk/arm movement, alternative grasping strategy
**2**	**Task is completed with modifications**<br>E.g. takes help of the other hand to stabilize the glass in grasp/release/transport and/or to move the glass closer to the body in order to grasp/release, reaches the mouth with glass but unable to drink, spills water
**1**	**Task is only partly completed**<br>Reaches the glass but can't grasp and/or reach the mouth despite modifications<br>*OBS! Movement time and grasping will be scored as 0, for other task components only reaching will be scored*
**0**	**The task is not completed**<br>Can't reach the glass despite modifications *OBS! All task components will be scored as 0*

Drinking Task Assessment – DTA

Component score

Components	
**Movement time** (mean of 3 trials)	
3	≤ 7.0 seconds
2	7.1 to 9.0 seconds
1	9.1 to 19.9 seconds
0	≥ 20 seconds or task is not fully completed despite minor modifications (score 0-1 on global score)
**Movement smoothness**	
3	Smooth and fluid arm movement
2	Slightly unsmooth arm movement, but no stops
1	Clearly unsmooth arm movement, minor stops, but still performed in a sequential manner
0	Complete segmentation of arm movement, one or more complete stops
**Trunk displacement**	
3	No or almost no compensatory trunk displacement
2	Small to moderate compensatory displacement of the trunk (compared to the performance with the other arm)
1	Excessive compensatory displacement of the trunk
0	Don't reach the glass or moves the glass closer to body prior grasp/release
**Shoulder flexion**	
3	Adequate shoulder flexion, no compensatory shoulder girdle movement
2	Shoulder flexion is combined with slight to moderate increased compensatory shoulder girdle movement
1	Shoulder flexion is minimal and/or combined with excessive compensatory shoulder girdle movement
0	No shoulder flexion is observed or moves the glass closer to body prior grasp/release
**Elbow extension** from the starting position of 90° until reaching and grasping the glass	
3	Adequate elbow extension, no compensatory trunk displacement
2	Elbow extension is decreased (compared to the other arm)
1	Elbow extension is minimal, hardly observable
0	No elbow extension is observed or moves the glass closer to body prior grasp
**Grasping** cylindrical grasp with opposed thumb	
3	Adequate hand opening during grasp and release of the glass
2	Minor to moderate difficulty to open the hand/fingers, grasp and/or release the glass
1	Major difficulty (several attempts) or uses an alternative grasping strategy (not cylindrical grasp with opposed thumb)
0	Unable to grasp or release the glass with the affected hand alone

**Global score (0-5 points)**	
**Component score (0-18 points)**	
**Total score (0-23 points)**	
